# Beyond the Knife: Integrating Near-Infrared Fluorescence Imaging in Complex Gluteal Tumor Resection and Reconstruction

**DOI:** 10.7759/cureus.88432

**Published:** 2025-07-21

**Authors:** Fernando Dip, Rene Aleman, Mariano F Ramirez, Javier I Ghiselli, Alberto Rancati

**Affiliations:** 1 General Surgery Department, University of Buenos Aires, Buenos Aires, ARG; 2 Heart, Vascular, and Thoracic Institute, Cleveland Clinic Florida, Weston, USA

**Keywords:** fluorescence-guided surgery, indocyanine green, malignant chondroid syringoma, near-infrared imaging, oncologic surgery

## Abstract

Malignant chondroid syringoma (MCS) is an exceptionally rare cutaneous adnexal tumor arising from sweat gland epithelium. The standard treatment involves wide local excision with clear margins, frequently followed by regional lymphadenectomy and flap reconstructive surgery. In anatomically complex regions such as the gluteal area, achieving both oncologic safety and reliable soft-tissue coverage poses significant surgical challenges. In this context, fluorescence-guided surgery (FGS) has emerged as a valuable intraoperative adjunct. Utilizing indocyanine green (ICG), FGS enables real-time perfusion mapping and enhanced visualization of tissue vascularity, thereby improving flap planning and inset accuracy. This is the case of a 51-year-old female patient who underwent radical resection of a large gluteal MCS and ipsilateral lymphadenectomy, followed by advancement flap reconstruction guided by near-infrared (NIR) fluorescence imaging using the novel IC-Flow 2™ system (Diagnostic Green Ltd, Munich, Germany).

## Introduction

Chondroid syringoma, or mixed tumor of the skin, is a rare adnexal neoplasm arising from sweat glands, which are typically benign in nature. Its pathogenesis is not well understood but is thought to originate from pluripotent ductal epithelial cells. While most commonly found in the head and neck region, these tumors can also occur on the extremities. They are frequently diagnosed between the ages of 30 and 60 years, although pediatric presentations have been documented [1]. Accounting for only 0.01% of all primary skin tumors, chondroid syringomas are frequently misdiagnosed due to their nonspecific clinical appearance and are often mistaken for cysts or other benign lesions [2]. Histological diagnosis reveals characteristic nests of basaloid epithelial cells within a chondroid or myxoid stroma [3]. Complete surgical excision is curative in most cases, with recurrence rates ranging from 2% to 9% when margins are inadequate. Malignant chondroid syringoma (MCS) is an exceptionally rare and aggressive variant, with only 51 cases reported. Lesions larger than 5 cm may warrant more aggressive treatment, with wide local excision remaining as the preferred approach [1]. Soft-tissue reconstruction using advancement flaps offers a practical solution for defect coverage following wide local excision. These flaps allow straightforward planning and reliable closure, particularly where secondary graft-site management is required [4-6]. Increasingly, fluorescence-guided surgery (FGS) using indocyanine green (ICG) has supported both oncologic and reconstructive goals. By leveraging near-infrared (NIR) imaging, FGS enhances margin assessment and real-time perfusion evaluation, thus improving surgical precision while preserving functional outcomes [7-9]. This report presents a rare case of MCS managed with radical excision and advancement flap reconstruction, utilizing ICG-based NIR imaging to guide perfusion assessment intraoperatively. The case underscores the clinical relevance of integrating FGS in complex cutaneous oncology.

## Case presentation

**Figure 1:**
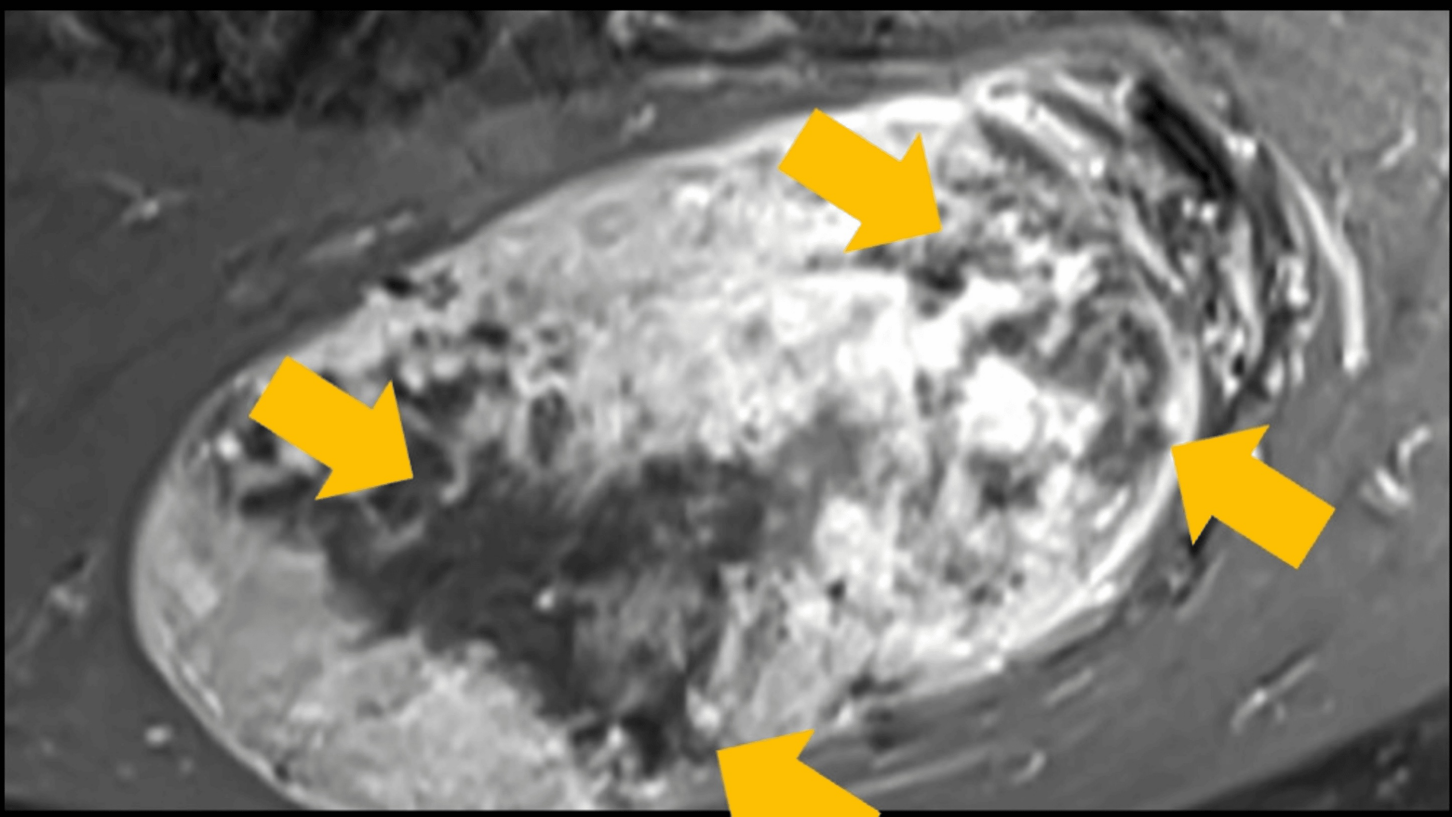
MRI of a large gluteal mass MRI displaying a large exophytic gluteal mass MRI: magnetic resonance imaging

**Figure 2:**
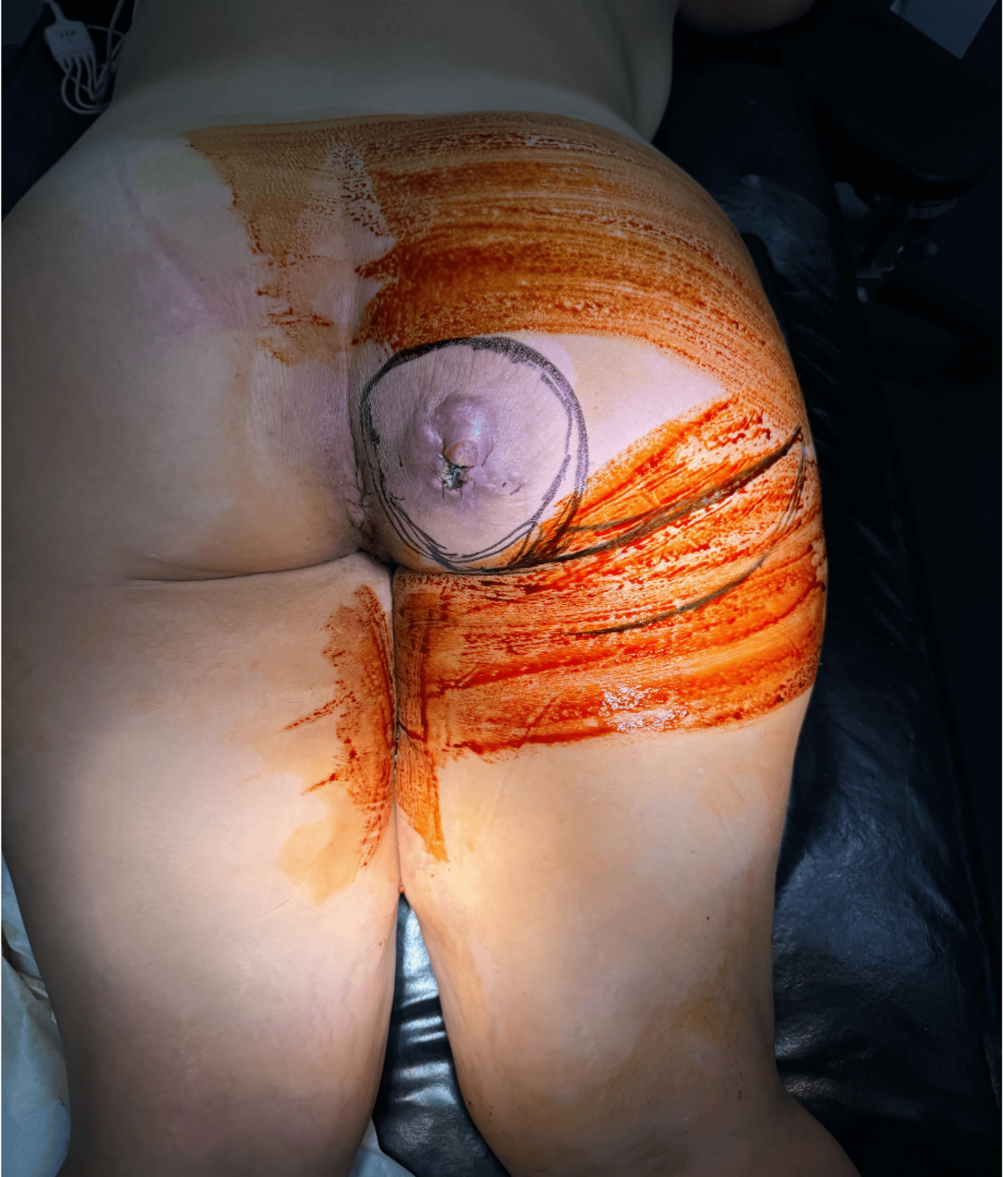
Right gluteal lesion prior to radical excision The exophytic mass was approximately 10 cm in circumference with a purulent discharge

**Figure 3:**
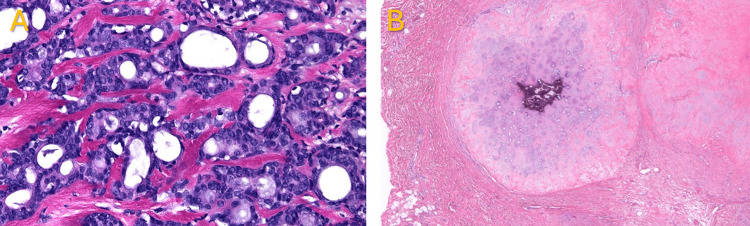
Tumor histopathology Malignant chondroid syringoma with epithelial tumor islands and chondroid mixed areas and epithelial cords with cubicle-lined ducts. A, 40× magnification; B, 400× magnification

This is the case of a 51-year-old female patient with a medical history of hypertension, obesity, type 2 diabetes mellitus, and Roux-en-Y gastric bypass performed seven years prior to this consult. The patient presented with a three-year history of a progressively enlarging right gluteal mass with no significant symptoms (Figure 1). Initially misdiagnosed and percutaneously drained as an abscess at an outside facility, physical examination revealed a firm, erythematous, exophytic mass, approximately 10 cm in circumference, with purulent discharge, fixed to the underlying deep tissues (Figure 2). Palpable right inferior gluteal lymphadenopathy raised suspicion for regional metastatic disease. Magnetic resonance imaging (MRI) was obtained to assess local invasion and the extent of the mass. An initial biopsy of the gluteal lesion, along with fine-needle aspiration of the inguinal lymph nodes, demonstrated malignant epithelial cells. Histopathological evaluation confirmed the diagnosis of a malignant chondroid syringoma (mixed cutaneous adnexal neoplasm) tumor, characterized by eccrine differentiation and chondroid stroma (Figure 3). Staging computed tomography (CT) with intravenous contrast ruled out distant metastatic disease. The patient subsequently underwent a radical wide excision of the gluteal tumor with ipsilateral inguinal lymphadenopathy and sliding flap reconstruction. The patient has provided verbal and written consent prior to intervention.

Surgical technique

The procedure was carried out with the standard radical tumor resection technique, performed under general anesthesia. ICG-mediated NIR fluorescence imaging, facilitated by the IC-Flow 2™ device (Diagnostic Green Ltd, Munich, Germany), was seamlessly integrated to provide dynamic vascular mapping and real-time perfusion assessment. This advanced guidance directly informed the subsequent reconstructive phase, leading to the precise execution of the sliding flap reconstruction and ensuring optimal tissue viability within the excised tumor defect.

**Figure 4:**
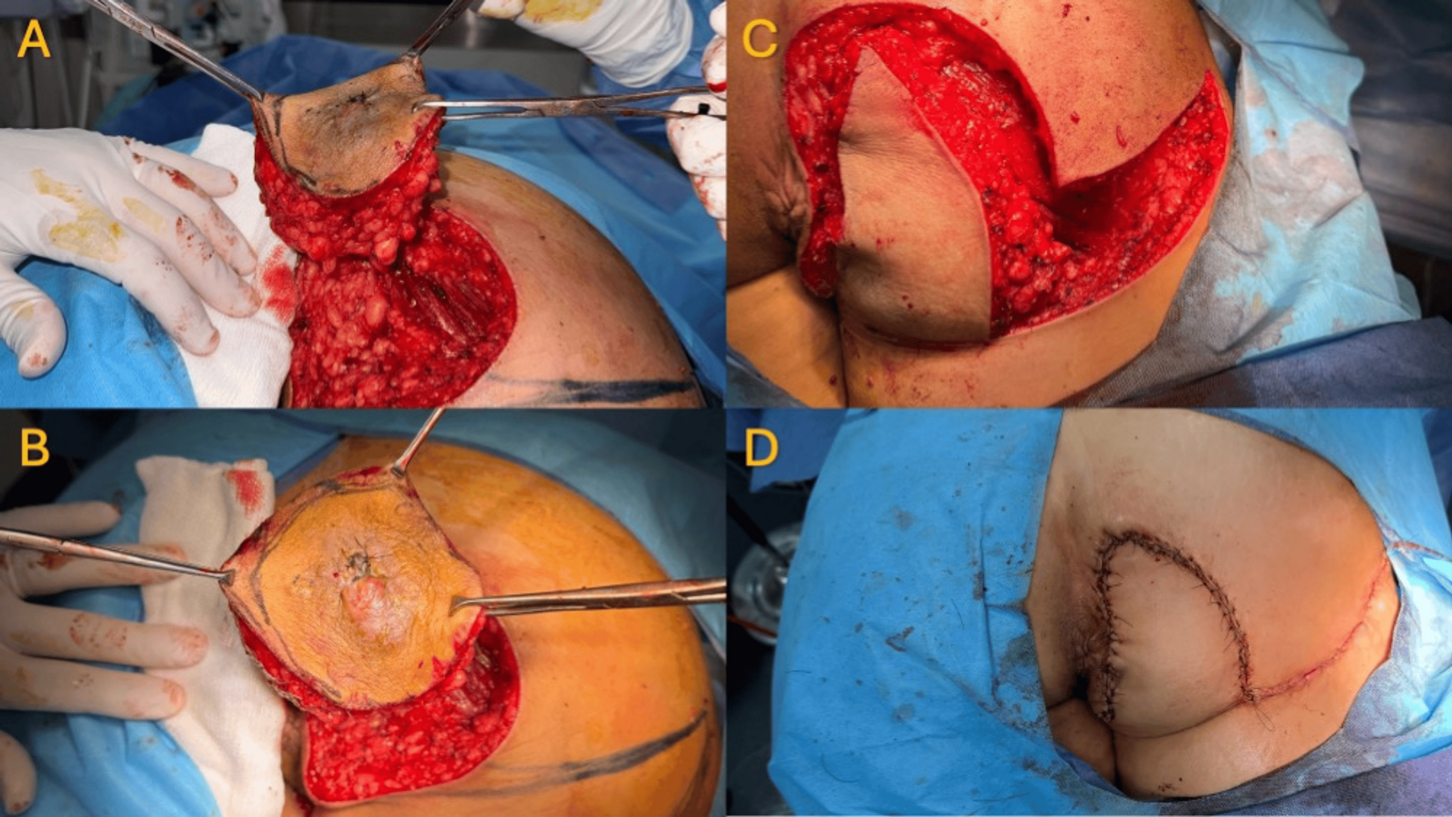
Radical excision and reconstruction Left (A-B): Free margin, radical excision of a right gluteal MCS. Right (C-D): Advancement flap reconstruction of a large soft-tissue defect post-tumoral excision MCS: malignant chondroid syringoma

Wide local excision of the gluteal mass resulted in an en bloc resection specimen measuring 9×12×6 cm, partially covered with overlying skin. Gross examination revealed a lobulated, firm, whitish neoplasm with central necrosis involving approximately 20% of the tumor volume and an associated cutaneous ulceration. The tumor measured 6×5×3 cm and was histopathologically confirmed as a malignant chondroid syringoma, characterized by the presence of eccrine differentiation and chondroid stromal features. Microscopic evaluation demonstrated perineural invasion and lymphovascular permeation. However, all surgical margins were negative. The closest margin, located at the superior and deep aspects, measures 2.5 cm. The ipsilateral inguinal lymphadenectomy specimen measured 11×7×6 cm. Histological analysis revealed metastatic carcinoma in three of eight dissected lymph nodes, with extranodal extension into the surrounding pre-vascular adipose tissue (Figure 4).

**Figure 5:**
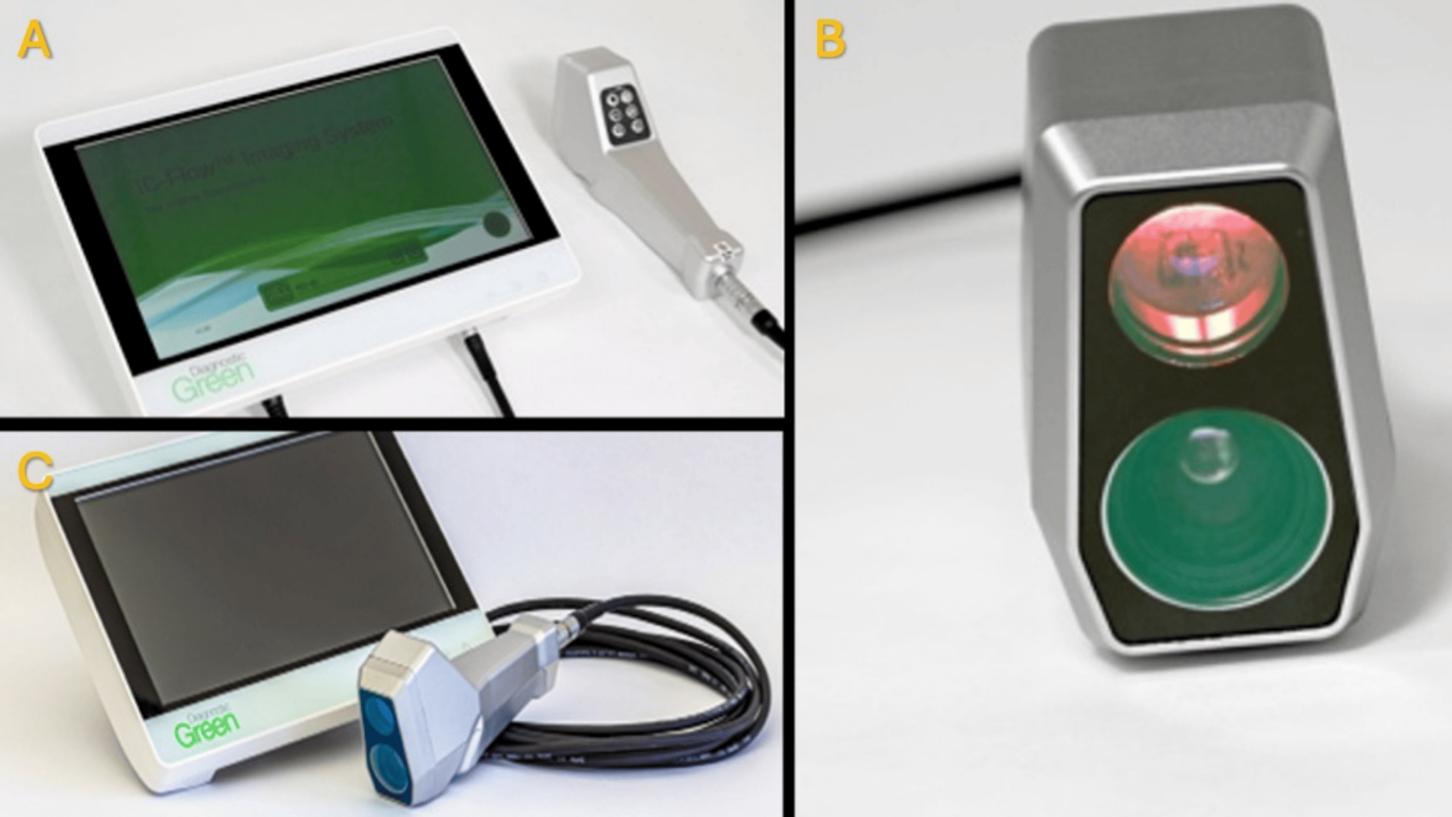
IC-Flow 2™ device IC-Flow 2™ device for indocyanine green near-infrared imaging. Device images provided by Diagnostic Green. Reproduced with permission from ©2025 Diagnostic Green. (A and C) Screen display and handheld device. (B) Near-infrared handheld device

The reconstruction of the resulting soft-tissue defect was achieved using a 20 cm fasciocutaneous advancement flap with medial rotation. The flap was inset under minimal tension, with meticulous multilayer closure to preserve contour and minimize dead space (Figure 4). The intraoperative assessment of flap viability was performed using NIR fluorescence angiography following the peripheral administration of 3 mL (7.5 mg) of ICG. Imaging was conducted using the IC-Flow 2™ platform (Figure 5), with angiography completed within 30-60 seconds of ICG injection during gluteal reconstruction (Video 1).

**Video 1:** Fluorescence angiography Intraoperative assessment of an advancement flap in a right gluteal reconstruction. Flap is visualized in contrast mode, where the white light highlights the perfusion and overall viability of the inset flap

**Figure 6:**
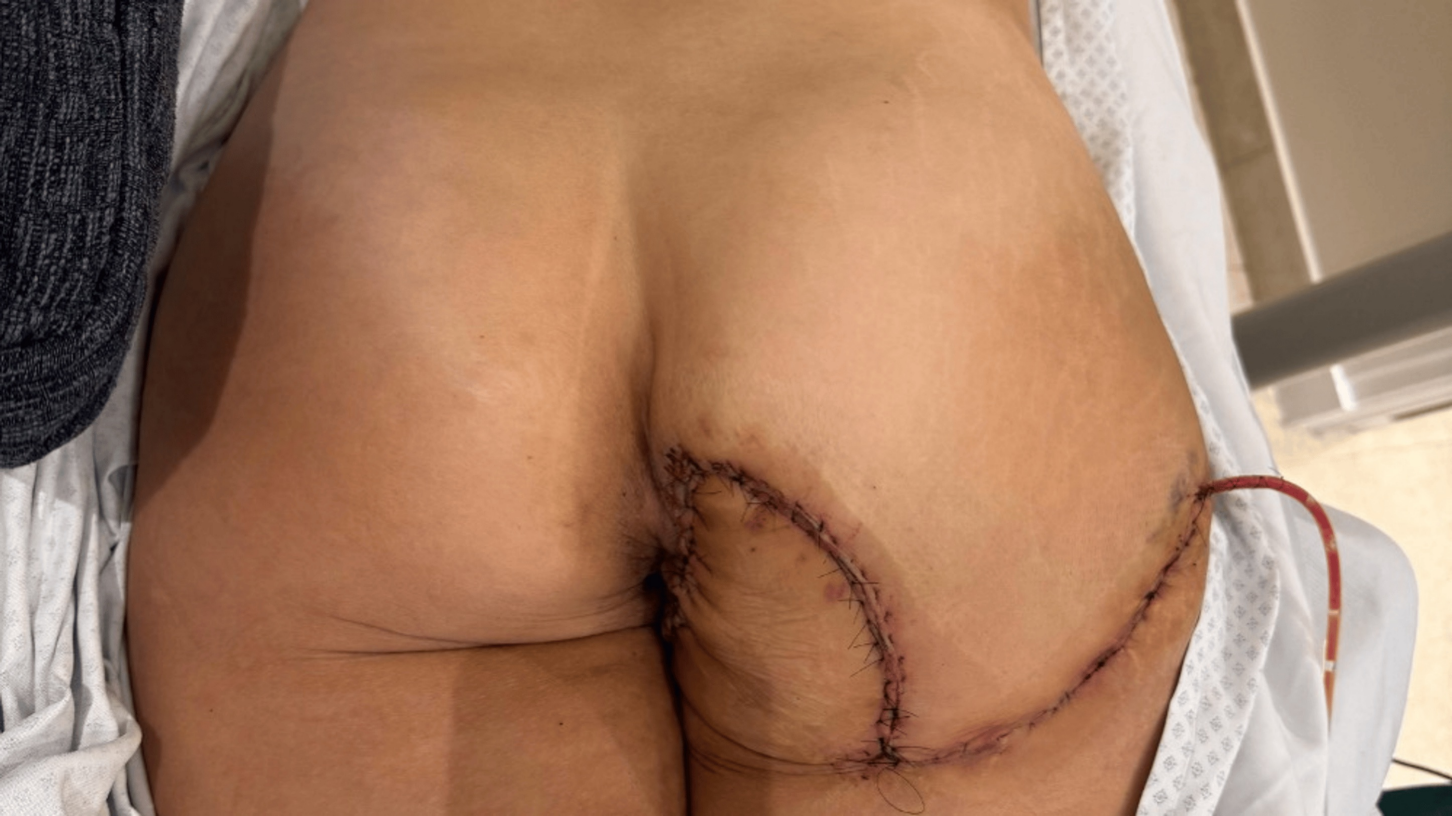
Postoperative right gluteal reconstruction Postoperative status of an advancement flap following malignant chondroid syringoma excision

The dynamic visualization of perfusion revealed immediate, homogeneous bright fluorescence throughout the cutaneous and subdermal vascular plexus of the flap, confirming robust perfusion and viability. No areas of hypoperfusion were identified. While diminished signal intensity may be indicative of compromised vascularity to guide adjustments in surgical conduct, none were required in this case. The surgical site was policed for bleeding to achieve complete hemostasis, and a closed-suction drain was placed in the deep wound bed. The patient tolerated the procedure well and was discharged on postoperative day 3, with no evidence of wound complications or flap compromise during early follow-up (Figure 6).

Postoperative course

The patient was discharged in stable condition with detailed instructions for outpatient follow-up, including ongoing wound care and close monitoring of flap viability. At the one-month follow-up visit, there were no signs of tissue necrosis or pain. The patient demonstrated preserved functional mobility, which continued to improve with adherence to a structured physical therapy program.

## Discussion

Malignant chondroid syringoma (MCS) is an exceptionally rare cutaneous adnexal neoplasm, with few cases documented in the literature. Due to its rarity, management must be approached strategically to prevent disease progression and improve outcomes. Surgical excision remains the mainstay of treatment, irrespective of tumor location. Prognostically, untreated MCS carries a reported mortality rate of up to 18%, with a metastasis rate of approximately 35%. This highlights the need for vigilant surveillance for local recurrence, nodal involvement, and distant spread. Notably, lesions arising on the extremities demonstrate a markedly higher metastatic potential, with a 65.4% rate, underscoring the importance of wide, oncologically sound resection [1]. Post-excisional reconstruction poses both functional and aesthetic challenges, particularly in anatomically complex regions. Advancement flaps offer a reliable option for defect closure, allowing for optimal tension distribution, scar concealment along cosmetic subunits, and the preservation of contour [5,10,11]. However, despite their versatility, partial flap necrosis rates range from 1% to 13.7%, while total necrosis may reach 10.3% [12]. To mitigate these risks, ICG-mediated FGS has emerged as a valuable adjunct in flap viability assessment. As a safe, feasible, and noninvasive tool, NIR imaging enhances the intraoperative visualization of perfusion [9]. This case illustrates how integrating ICG-based NIR imaging into oncologic and reconstructive workflows can strengthen surgical decision-making and improve outcomes, even in rare and complex tumors such as MCS.

The rarity of MCS far exceeds that of its benign counterpart. Histological criteria for malignancy remain somewhat imprecise, as tumors lacking overt cytologic atypia have demonstrated metastatic potential and recurrence [13]. However, features such as nuclear atypia, cellular pleomorphism, increased mitotic activity, infiltrative growth patterns, satellite nodules, necrosis, and evidence of vascular or perineural invasion are generally considered diagnostic indicators of malignancy. Wide local excision remains the primary treatment modality for MCS. Radiation therapy (RT) has been employed in only 6% of reported cases, typically as an adjunct. Amputation has been necessary in another 6% of cases involving limb MCS. Mohs micrographic surgery has had limited utility, reported in just 2% of cases. For local recurrence, wide local excision has been the most frequently applied strategy, used in up to 58% of patients, with adjunct RT administered in 25% of these cases. In the setting of metastatic disease, a combination of surgical excision, RT, and chemotherapy has been employed [1,13]. The present case demonstrated hallmark histopathological features of malignancy, which were managed with wide local excision and histologically confirmed negative margins. At six-month follow-up, the patient remains recurrence-free, though ongoing surveillance is warranted given the absence of standardized guidelines.

Postoperative soft-tissue defects often necessitate surgical reconstruction. When primary closure is not feasible, local advancement flaps serve as a highly effective strategy, preserving native tissue perfusion while avoiding the complexity of microsurgical techniques. These flaps recruit tissue adjacent to the wound and typically rely on the subdermal vascular plexus for perfusion, making careful design essential. Maintaining appropriate length-to-width ratios is critical to ensure viability and avoid complications such as ischemia or venous congestion. Success with advancement flaps hinges on meticulous planning. Incisions should be aligned with relaxed skin tension lines or aesthetic unit boundaries to optimize both healing and cosmetic outcomes. Proper tension distribution is equally important to minimize the risk of dehiscence and flap necrosis. Their versatility across anatomical regions reinforces their value in restoring function and form, particularly in challenging reconstructions [14,15]. In this case, given the large gluteal defect and the complexity of surrounding anatomical structures, an advancement flap was the most appropriate reconstructive option to minimize morbidity [5]. Specifically, advancement flaps in perineal and pelvic reconstruction have demonstrated 100% survival rates, although minor complications such as surgical site infection, dehiscence, and drainage-related reintervention have been reported [4]. Flap necrosis, frequently resulting from venous congestion, continues to be the most prevalent complication in reconstructive flap procedures [16]. Although no such events occurred in this case, the authors attribute this outcome to the use of intraoperative NIR fluorescence imaging, which provided real-time assessment of flap perfusion and guided precise flap inset, potentially reducing the risk of failure.

Indocyanine green (ICG)-mediated FGS has gained increasing traction as an intraoperative adjunct to improve outcomes in oncologic procedures. Ensuring patency of micro- and macrovascular anastomoses is critical for successful flap inset, and the early detection of vascular compromise remains essential for flap salvage. When used alongside clinical assessment, adjunctive perfusion monitoring can significantly increase the success rate of reconstructive procedures [9]. Fluorescence angiography has advanced considerably, becoming more accessible, cost-effective, and user-friendly in routine surgical practice [17-19]. In autologous breast reconstruction using free flaps, ICG angiography has demonstrated utility in reducing postoperative fat necrosis by allowing the intraoperative visualization of marginally perfused tissue, enabling the timely excision of ischemic zones [20,21]. Despite its widespread use in free flap surgery, reports of ICG imaging in advancement flap procedures remain limited [22-24]. Given the restricted tissue available for transfer in these flaps, real-time perfusion assessment is crucial, particularly when reconstruction involves the coverage of high-risk or functionally critical regions. In this case, the IC-Flow 2™ device was instrumental in assessing flap viability and minimizing the risk of vascular compromise during inset. To the best of the authors’ knowledge, this represents the first reported use of NIR perfusion imaging in reconstructive surgery following the resection of a large MCS. While the results presented are promising, further studies with larger cohorts are necessary to validate these findings and establish standardized protocols for the integration of FGS in complex oncologic reconstructions.

## Conclusions

This case highlights the clinical utility of NIR FGS using ICG in the management of MCS with complex reconstruction. Real-time perfusion imaging with the IC-Flow 2™ system proved to be a valuable adjunct in ensuring flap viability, enhancing intraoperative decision-making, and optimizing reconstructive outcomes. As surgical oncology increasingly prioritizes precision and preservation, the integration of ICG-based imaging offers a practical, evidence-supported tool with the potential to improve both oncologic safety and reconstructive success.
